# Short-term flooding increases CH_4_ and N_2_O emissions from trees in a riparian forest soil-stem continuum

**DOI:** 10.1038/s41598-020-60058-7

**Published:** 2020-02-21

**Authors:** Thomas Schindler, Ülo Mander, Katerina Machacova, Mikk Espenberg, Dmitrii Krasnov, Jordi Escuer-Gatius, Gert Veber, Jaan Pärn, Kaido Soosaar

**Affiliations:** 1 Department of Geography, Institute of Ecology & Earth Sciences, University of Tartu, Tartu, Estonia; 2 Department of Ecosystem Trace Gas Exchange, Global Change Research Institute of the Czech Academy of Sciences, Brno, Czech Republic; 3 Department of Plant Physiology, Estonian University of Life Sciences, Tartu, Estonia; 4 Chair of Soil Science, Estonian University of Life Sciences, Tartu, Estonia

**Keywords:** Biogeochemistry, Environmental sciences

## Abstract

One of the characteristics of global climate change is the increase in extreme climate events, e.g., droughts and floods. Forest adaptation strategies to extreme climate events are the key to predict ecosystem responses to global change. Severe floods alter the hydrological regime of an ecosystem which influences biochemical processes that control greenhouse gas fluxes. We conducted a flooding experiment in a mature grey alder (*Alnus incana* (L.) Moench) forest to understand flux dynamics in the soil-tree-atmosphere continuum related to ecosystem N_2_O and CH_4_ turn-over. The gas exchange was determined at adjacent soil-tree-pairs: stem fluxes were measured in vertical profiles using manual static chambers and gas chromatography; soil fluxes were measured with automated chambers connected to a gas analyser. The tree stems and soil surface were net sources of N_2_O and CH_4_ during the flooding. Contrary to N_2_O, the increase in CH_4_ fluxes delayed in response to flooding. Stem N_2_O fluxes were lower although stem CH_4_ emissions were significantly higher than from soil after the flooding. Stem fluxes decreased with stem height. Our flooding experiment indicated soil water and nitrogen content as the main controlling factors of stem and soil N_2_O fluxes. The stems contributed up to 88% of CH_4_ emissions to the stem-soil continuum during the investigated period but soil N_2_O fluxes dominated (up to 16 times the stem fluxes) during all periods. Conclusively, stem fluxes of CH_4_ and N_2_O are essential elements in forest carbon and nitrogen cycles and must be included in relevant models.

## Introduction

Greenhouse gases (GHG), in particular, methane (CH_4_) and nitrous oxide (N_2_O) contribute 16% and 6% to global warming, respectively^1^. In addition, N_2_O is a dangerous stratospheric O_3_ layer depleting agent^2^. Due to the increasing emissions, both gases have high radiative forcing potential. In principle, terrestrial biosphere may be seen as a net source of GHG to the atmosphere^3^. Temperate as well as tropical forest soils (in general) seem to be a central natural emitting source of N_2_O, on the one hand, a natural sink of CH_4_ on the other^4–9^. Flux estimations of N_2_O and CH_4_ in forest systems are mainly based on studies of forest soil measurements, usually excluding exchange potential of vegetation^5,7,10^. Nevertheless, investigations on GHG fluxes from plants in wetland or riparian ecosystems show that plants, especially trees, can be essential sources of CH_4_ and N_2_O^9,11–13^. However, recent studies uncover the relevance of tree stem surfaces playing an important role in understanding GHG dynamics in different forest ecosystems^8,9,14^.

Grey alder (*Alnus incana* (L.) Moench)) is a fast-growing, pioneer tree species with excellent potential for short-rotation forestry in the Northern hemisphere^15–18^. Due to the symbiotic *Frankia* bacteria which fix atmospheric nitrogen, alder forests are important nitrogen sequestering ecosystems^19,20^. Decomposition of nutrient-rich alder litter improves soil properties, in particular, the carbon:nitrogen (C:N) ratio^21–24^, which alters the microbial activity in the soil and affects the production and consumption of CH_4_ and N_2_O in the soil^25^.

CH_4_ is produced under anaerobic conditions in a water-saturated environment by methanogenic archaea and can be oxidised by aerobic or anaerobic methanotrophs. N_2_O, on the other hand, is a natural product of several N turnover processes (e.g. nitrification, denitrification)^10,26^. Even if both gases release into the atmosphere by gas diffusion at the soil surface, trees may contribute to ecosystem GHG exchange by i) gas uptake from soil via their root system, transport into the aboveground tree tissues and emission into the atmosphere; ii) uptake of CH_4_ and N_2_O from the atmosphere, or iii) alternation of gas turnover processes in adjacent soils^12,27,28^_._

In reaction to stressing factors, for instance, a natural or even artificial creation of anaerobic soil conditions due to flooding events, biosynthetic processes like production and consumption of CH_4_ and N_2_O and their respective transportation may become modified and, thus, may lead to a change of the potential trace gas emissions^29–31^. Importantly, plant species react differently, depending on their anatomical and physiological predisposition/adaptation to stresses, soil characteristics^32^, seasonal effects, temperature^33^, and intensity of the stressing event in general^26^.

Nevertheless, field experiments, investigating CH_4_ and N_2_O fluxes in riparian tree communities exposed to environmental stress are scarce. Thus, a large-scale forest manipulation experiment was conducted in a grey alder forest in summer 2017. The main objective of the FluxGAF (“Biogeochemical **Flux**es in **G**rey **A**lder **F**orest”) campaign was to investigate the response of the forest ecosystem to heavy overland flow. The objective was to understand the biochemical process and flux dynamics in the soil-tree-atmosphere continuum related to ecosystem CH_4_ and N_2_O turnover and exchange. Therefore, we quantified CH_4_ and N_2_O fluxes from stems of grey alder and adjacent soil in response to an artificial flooding event. The trace gas fluxes were measured on mature trees in an alder forest in Estonia together with forest floor CH_4_ and N_2_O fluxes, and a variety of environmental parameters (including (micro)meteorological, soil and atmospheric parameters). The study site consisted of two plots: a flooded plot (FP), where 55–70 m³ of water per day was applied for two weeks in summer 2017, and a control plot (CP). The study period was divided into three periods: pre-experimental (July 24^th^–August 7^th^), experimental (mimicking flooding; August 8^th^–21^st^) and post-experimental (August 22^nd^–September 4^th^).

We hypothesize that: (1) due to the flooding, emission of methane and nitrous oxide from both the soil and tree stems will increase while the lower parts of stems will have higher emission, (2) flooding will change the proportion of soil and stem fluxes in overall emissions while the dynamics are different for CH_4_ and N_2_O.

## Results

### Soil physicochemical conditions and characteristics of the tree stand

**Figure 1:**
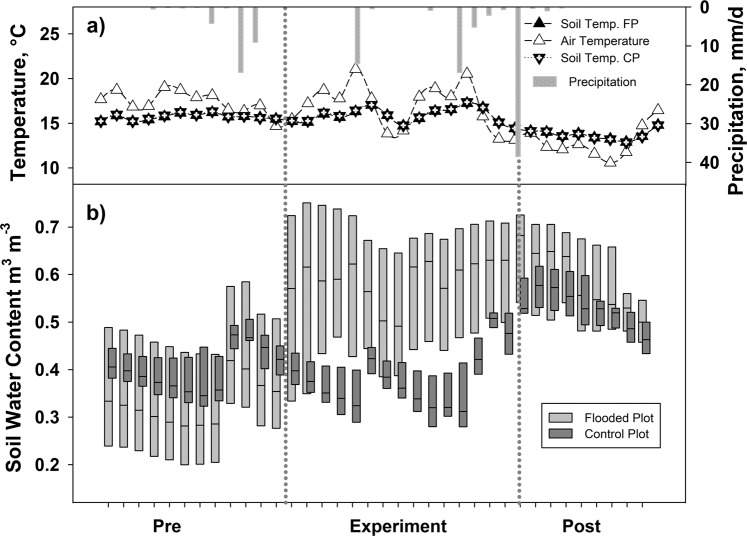
Environmental parameters at pre-experimental (Pre), experimental (Experiment) and Post-Experimental (Post) period: (**a**) soil temperature at 5 cm depth; averaged per each soil chamber at the flooded plot (FP) and control plot (CP), air temperature, and the daily sum of precipitation (n = 36), (**b**) Soil water content at FP and CP. Boxes indicate median, 25^th^ and 75^th^ percentile values. Each data point is the value at 12:00 per day.

The study period was characterised by mean air temperature (mean ± standard deviation) of 19.9 ± 2.1 °C, and soil temperature at a soil depth of 5 cm at the flooded and control plot, 15.1 ± 0.6 and 15.3 ± 0.6 °C, respectively (Fig. 1a). No significant differences were detected in Soil Water Content (SWC) between flooded and control plots in the pre-experimental period, although SWC was significantly higher in the flooded plot during the experimental (p < 0.001) and post-experimental periods (p < 0.05) (Fig. 1b and Supplementary Table S1). Single rain incidents occurred and one at the beginning of post-experimental period also increased SWC in the control plot (Fig. 1a). These trends were also reflected in the water table (Supplementary Fig. S1).

Tree stand density was approximately 1500 trees per hectare and tree height was 19.2 ± 1.4 m in both studied plots. In addition, tree diameter at 1.3 m height showed no significant difference between FP and CP and was approximately 0.17 ± 0.03 m on both sites.

**Figure 2:**
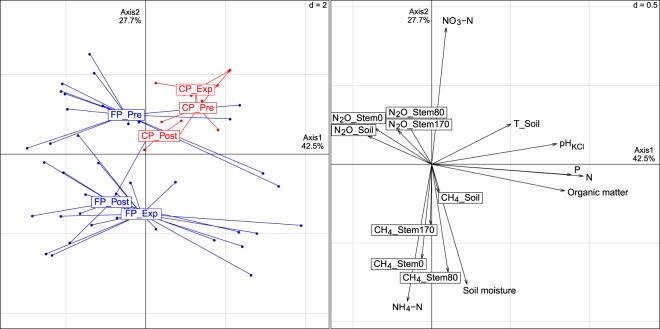
Characteristics of physico-chemical and gas flux parameters in the flooded (FP) and control plots (CP) of pre-experimental (Pre), experimental (Exp), and post-experimental (Post) periods. The principal components analysis (PCA) is based on imputed data set (n = 55). Abbreviations: T_Soil–soil temperature; Stem0, Stem80, and Stem170 denote measurements at three stem heights of 10, 80 and 170 cm above the ground. PCA based on real data set (n = 31) is shown in Supplementary Fig. S3.

Experimental (FP_Flooding) and post-experimental (FP_Post) periods of the flooded plot were clearly distinct from pre-experimental period of the flooded plot and all periods of the control plot (p < 0.05; Fig. 2). Pre-experimental period of the flooded plot (FP_Pre) was different from the experimental period of the control plot (CP_Flooding; p < 0.05). The flooded plot was characterised by higher NH_4_ + levels during the experimental (p < 0.001) and post-experimental period (p < 0.05), and lowered NO_3_ − values during the experimental period (p < 0.001, Supplementary Fig. S2). pH, phosphorous (P), nitrogen (N), organic matter and soil temperature values showed no significant differences between the two plots in different periods. Soil bulk density varied between 0.77 and 0.96 and was not significantly different between studied plots.

### Fluxes of CH_4_ and N_2_O and their relation to environmental factors

Different patterns emerged for CH_4_ and N_2_O emissions in the studied plots and their periods (Fig. 2). No significant differences were detected in CH_4_ emissions from soil and 170 cm stem level between flooded and control plots in the pre-experimental period, although CH_4_ emissions were significantly higher from soil and all stem levels in the flooded plot during the experiment (p < 0.05 for soil, p < 0.001 for all stem levels) (Supplementary Table S1). Moreover, CH_4_ emissions were significantly higher from the soil and stems at 10, 80 and 170 cm level in the flooded plot compared to the control plot during the post-experimental period (p < 0.001 in all cases).

N_2_O emissions from the soil and different stem heights showed more controversial results (Supplementary Table S1). Very few significant differences in N_2_O emission appeared between flooded and control plots before and during the experiment. However, N_2_O emissions from all stem heights were significantly lower in the flooded plot compared to the control plot during the post-experimental period (p < 0.001 in all cases).

Soil NO_3_^−^ was positively and NH_4_^+^ was negatively related to N_2_O emissions from soil and different stem heights (Fig. 2). In addition, soil moisture was negatively related to N_2_O emissions, whereas it showed positive relationships to CH_4_ emissions from both the soil and stems. Soil temperature, pH, P, N, and organic matter were almost perpendicular to the CH_4_ and N_2_O flux vectors, indicating little or no correlations.

**Figure 3:**
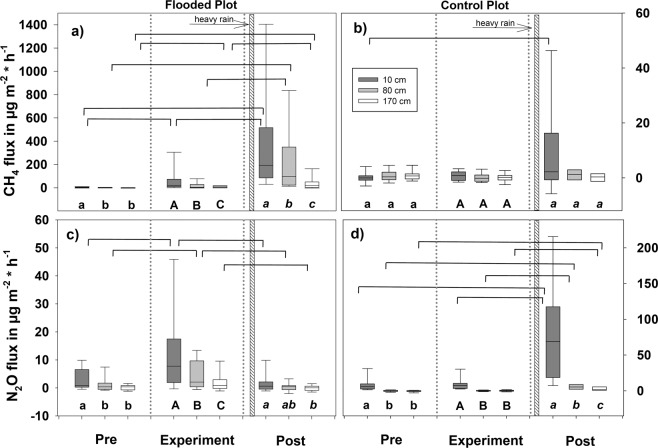
Stem fluxes of CH_4_ (**a**,**b**) and N_2_O (**c**,**d**) during the study period at 10, 80 and 170 cm heights (µg m^−2^ h^−1^). n (**a,c**) =54/36/36/58/39/39/36/24/24; n (**b,d**) =18/12/12/21/14/14/12/8/8. The stem fluxes are calculated for soil surface area equivalent. Letters below bars (small letters for pre-experimental, capital letters for experimental, and small italic letters for post-experimental period) indicate statistically significant differences in fluxes of each stem height among the periods, differences in fluxes between adjunct periods are marked with brackets (p < 0.05). Notice the scale difference in the flooded and control plots. The positive fluxes indicate emission, the negative fluxes gas uptake. The solid line within each box marks the median value, box boundaries the 25^th^ and 75^th^ percentiles, whiskers the 10^th^ and 90^th^ percentiles.

**Figure 4:**
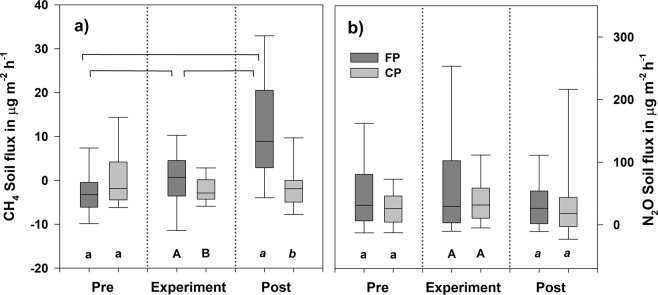
CH_4_ (**a**) and N_2_O (**b**) fluxes from the soil surface at the study period in µg m^−2^ h^−1^. From left to right n (**a,b**) =36/19/49/28/27/14/36/19/50/28/29/13. The letters below the bars (small letters for pre-experimental, capital letters for the experimental, and small italic letters for the post-experimental period) indicate statistically significant differences (p < 0.05). The positive fluxes indicate emission, the negative fluxes gas uptake. The solid line within each box marks the median value, box boundaries the 25^th^ and 75^th^ percentiles, whiskers the 10^th^ and 90^th^ percentiles.

Both soil and stem surfaces were net emitters of CH_4_ and N_2_O (Figs. 3 and 4). At the flooded plot, we observed an increase (p < 0.05) of stem CH_4_ fluxes at 10 cm from the pre- to the post-experimental period (Fig. 3a). A significant decline (p < 0.05) along the overall vertical stem profile was found during and after the experiment. On the control plot, there was significant difference at the 10 cm level before and after the experimental period only (Fig. 3b).

At the flooded plot, N_2_O fluxes from the 10 and 80 cm level of stems increased significantly with the flooding experiment and declined afterwards (p < 0.001). Fluxes from the 170 cm level decreased significantly during the post-flooding period only (p < 0.001). The N_2_O fluxes showed a diminishing trend with tree height (Fig. 3c).

At the 10 cm level in the control plot, we observed tendentially higher N_2_O fluxes without a general decrease with increasing stem height (p > 0.1). Nevertheless, the intensive rain forced occasional peaks with significant effect at the post-experimental period (Fig. 3d).

The soil CH_4_ fluxes increased significantly (p < 0.001) from light consumption (−0.2 ± 1.7 µg m^−2^ h^−1^, mean ± std. err.) before the experimental to 12.8 ± 2.1 µg m^−2^ h^−1^ at the post period (Fig. 4a). N_2_O fluxes from soil did not differ significantly (p > 0.2) either between the periods nor plots (Fig. 4b).

**Figure 5:**
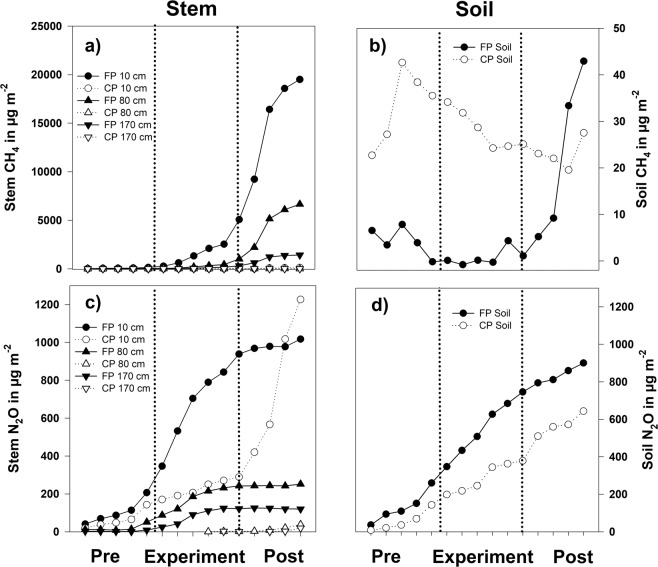
Cumulative daily mean stem (10, 80, 170 cm vertical profile) and soil fluxes of CH_4_ and N_2_O at the pre-experimental (pre), experimental, and post-experimental (post) periods. Notice the scale difference between the stem (**a**) and soil fluxes (**b**) of CH_4_.

Cumulative values of mean flux for each sampled day (Fig. 5) clearly demonstrate the flooding-induced dominance of CH_4_ fluxes from tree stems over soil emissions: the difference is up to 400 times. Furthermore, the vertical decrease in both CH_4_ and N_2_O emissions along the stem profile was remarkably enhanced by the flood. Methane fluxes from the soil, on the other hand, weighed in after the experiment.

Cumulative N_2_O fluxes responded positively to the artificial flooding and remained almost stable afterwards. The soils showed almost linear rise in cumulative emissions.

### The relative contribution of the stems and soil to the CH_4_ and N_2_O fluxes

**Figure 6:**
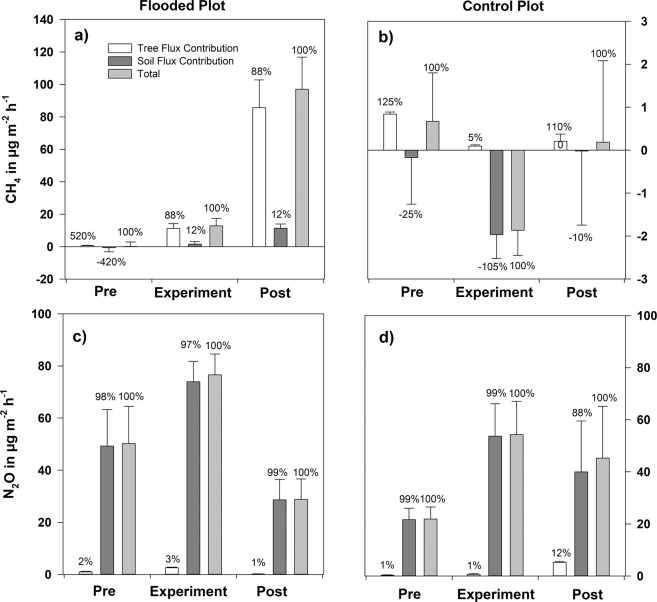
Contributing fluxes at the pre-experimental (pre), experimental, and post-experimental (post) periods from the stems and soil in µg m^−2^ h^−1^, scaled to a unit of the ground area of forest. Positive fluxes indicate emission, negative fluxes gas uptake. The boxes represent fluxes as means ± standard error. n (**a**,**c**) = 45/36/58/50/36/29, n (**b**,**d**) =15/22/21/28/12/13. The contributions of the stem to soil fluxes are expressed as percentages of the sum of stem and soil fluxes.

Stem fluxes were upscaled to unit of ground area of forest and compared with related soil fluxes (Fig. 6). At the flooded plot, CH_4_ emissions from tree stems dominated 7 times more with up to 88% contribution but small uptake rates from soil surfaces balanced with stem emissions at the control area (Fig. 6a,b).

Stems and soils were N_2_O emitters while no consumption was observed. Soil dominated N_2_O flux ratio on both plots with up to 99%, accordingly up to 145 times more. However, stem fluxes contributed 12% at the control plot’s post-experimental period (Fig. 6c,d).

## Discussion

### The relevance of the experimental set-up

The choice of the experimental area depended on many environmental factors. One of them was a slight slope which allowed slow movement of the added water and, most importantly, did not influence groundwater level and quality on the adjacent control plot (Supplementary Fig. S4). The impact of the slope and a 1 m high dyke, which separated the flooded and control plots, can be seen in the similar characteristics of FP and CP during the pre-experimental period (Supplementary Table S1, Figs. 4 and 5).

### Main controllers of CH_4_ and N_2_O fluxes

Methane is produced in anoxic soils and sediments, while well-drained soils act as a sink for atmospheric CH_4_ due to methane oxidation, both processes are controlled by different microorganisms. The main environmental factors controlling the CH_4_ emission in soils are the availability and quality of carbon, soil temperature and water content^34^. Since in our study area, the carbon content of the soil is not a limiting factor (Fig. 2), soil water content (water table) and temperature determine the most variation of CH_4_ fluxes in soil and stems (Fig. 2, Supplementary Table S1).

N_2_O is produced in the soil mainly via nitrification under aerobic conditions, where ammonia is oxidised, and by denitrification, which occurs under anaerobic conditions, where nitrate is sequentially reduced to nitrite, N_2_O and pure molecular nitrogen (N_2_)^35^. The gaseous nitrogen losses directly depend on soil nitrate content and soil moisture which affects oxygen availability in the soil^36^. Soils at a water content of 0.5–0.6 m^3^ m^–3^ emit the largest amounts of N_2_O^36^. This soil water content indicates the commonly known condition where both nitrification and denitrification contribute N_2_O^37,38^.

Under laboratory conditions, Unger *et al*.^31^ showed that alternating oxic/suboxic and anaerobic conditions, which are similar to our field study soil moisture, can coherently change NH_4_^+^ and NO_3_^–^ concentrations. Also, under laboratory conditions, Klemedtsson *et al*.^39^ showed the relationships between soil moisture and N_2_O production during nitrification and denitrification. Nevertheless, variation in N_2_O concentrations does not stringently correlate to variation of denitrifying activity. N_2_O can be produced by a range of organisms, further, denitrifiers such as bacteria or archaea may both produce and consume N_2_O. Nitrate may limit denitrification in forest ecosystems^40^. In our study, a decline of nitrate was coherent with the increase of ammonia in the soil (Supplementary Fig. S2).

In conclusion, the flash flooding significantly enhanced both nitrification and denitrification processes in the soil. N_2_O could be produced from both processes. Further, flooding can accelerate production of both CH_4_ and N_2_O that may lead to increased emissions from forest ecosystems.

### Comparison of results with outcomes from similar studies

Although our full-size flooding experiment during a reliable timeframe is the first attempt to measure both CH_4_ and N_2_O emissions from soil and tree stems simultaneously, there are analogous experiments conducted in laboratory conditions and/or in mesocosms. Rusch and Rennenberg^11^ considered an increase of N_2_O emissions from 3 years old black alder seedlings immediately after flooding had started but no effect on CH_4_ emissions but *vice versa* results after 40 days, in particular, N_2_O fluxes were below the detection limit, but CH_4_ increased enormously. Further, they found the efflux decrease with stem height from 0 to 2 m. On the other hand, Keppler *et al*.^41^ concluded after greenhouse experiments that even plants may produce CH_4_ *in situ*, covering 10–30% of the world’s total CH_4_ emissions. However, Terazawa *et al*.,^42^ concluded soil temperature and water table depth as possible environmental factors controlling stem CH_4_ emissions”. Mander *et al*.^43^, for instance, investigated the impact of flooding, using a pulsing groundwater level on GHG fluxes from the soil and found an increase of CH_4_ emissions but a decrease of N_2_O emissions after flooding. However, our field experiment on flooding the *Alnus incana* forest indicated a “chimney effect” similar to Rice *et al*.^44^, i.e. soil microbes produce CH_4_ which is transported via roots, stems, and leaves to be released to the atmosphere. Comparing to a mesocosm experiment with *Alnus glutinosa* seedlings under laboratory conditions conducted by Machacova *et al*.^12^ we found an increase of flooding-induced N_2_O stem emissions of up to 10 times (instead of a factor of 740 by Machacova *et al*.) but CH_4_ emissions in our experiment were up to 100 times higher. To compare with other studies, our flooding-induced CH_4_ emissions from alder stems were significantly higher, especially when comparing them with the soil fluxes. Pitz *et al*.^45^ measured methane fluxes from tree stems and soils along a habitat gradient and found mean stem CH_4_ emissions of 68.8 ± 13.0 (mean ± standard error), 180.7 ± 55.2 and 567.9 ± 174.5 g CH_4_-C m^−2^ h^−1^ for the upland, transitional and wetland habitats, respectively. In the same time, mean soil methane fluxes in the upland, transitional and wetland were −64.8 ± 6.2, 7.4 ± 25.0 and 190.0 ± 123.0 g CH_4_-C m^−2^ h^−1^, respectively.

Fluxes from other tree compartments can have an effect on CH_4_ and N_2_O balances. Machacova *et al*.^46^ show that mature Scots pine trees consistently emit CH_4_ and N_2_O from both stems and shoots. The shoot fluxes of CH_4_ and N_2_O exceeded the stem flux rates by 41 and 16 times, respectively. Therefore, further investigations at canopy level are very important.

Likewise, full-year investigations of gas emissions might change the results. For instance, Machacova *et al*.^47^ found a clear N_2_O emission peak from stems of boreal trees during the vegetation season/summer while there was no effect of soil water content on tree N_2_O fluxes in the vegetation season.

In future climate, the frequency of both flash floods and drought events is expected to be increased^48–51^. Although trees and other plants indicate the “forest ecosystem”, their role within gas cycles is somehow underestimated^12^. Therefore, understanding the capacity of ecosystems to adapt to such environmental modifications is the key to predict ecosystem responses to global change^52^.

## Conclusions

Our experimental flooding induced changes of water regime and consequent dynamics of CH_4_ and N_2_O pathways: emission of these gases from both soil and tree stem increased significantly. Thus, our first hypothesis has been supported. As well, the lower parts of stems showed higher emission compared with the higher positions. Likewise, the second hypothesis could be supported: we saw that flooding changed the proportion of soil and stem fluxes in overall emissions showing significantly different patterns for CH_4_ and N_2_O. Methane fluxes from tree stems were observed up to 100 times higher than related fluxes from soil chambers. In contradiction, nitrous oxide fluxes from the soil surface were up to 16 times higher than stem N_2_O fluxes close to the ground at the post-experimental period. Furthermore, the stems contributed up to 88% of CH_4_ emissions to the stem-soil continuum during the investigated period but soil fluxes dominated the N_2_O contribution during all periods when upscaling for forest area unit. A substantial link was shown between N_2_O fluxes from the stems and soil with soil water content and nitrogen availability as the main controlling factors.

Our results convince us that the N_2_O and CH_4_ exchange of riparian trees should be included in forest ecosystem GHGs budgets and forest-ecosystem process models. In addition, extreme climate events (flooding, drought) are significantly altering not only the soil fluxes, but also the stem fluxes. However, emission from tree stems of different species may vary significantly and further studies are needed for comprehensive flux estimates for different forest types.

## Material and Methods

### Site description and experimental design

The measurements were performed at the experimental site of Agali (58°17′N; 27°17′E) situated in eastern Estonia, 10 km west of Lake Peipus. The studied forest is a 40-year old hemiboreal *Filipendula* type grey alder (*Alnus incana (L.)* Moench) forest stand in a former agricultural Gleysol.

The artificial flooding was conducted in summer 2017 and was divided into three periods: a pre-experimental (July 24^th^–August 07^th^), experimental (August 8^th^–21^st^), and a post-experimental (August 22^nd^–September 4^th^) period. Within the forest, two experimental plots were established, a flooded plot (FP, 40 × 40 m), where water was pumped into using an irrigation pipe system mimicking the intensive-rain induced overland flow, and a control plot (CP, 20 × 20 m). The plots were separated by a 1 m high natural dyke preventing the spread of water into the control plot. Slight slope (1%) parallel to the dyke guaranteed isolated groundwater dynamics in the plots (Supplementary Fig. S4). Every experiment day, 55 to 70 m^3^ of pond water (pH 7.41, total C 76 mg/l, total N 1.8 mg/l, NH_4_^+^-N 0.79 mg/l, NO_3_^−^-N 32.6 µg/l) was applied to the FP by a fire truck. The total water amount of 875 m^3^ was distributed over 1,600 m² forest area, which is an equivalent to 547 mm precipitation. The long-term average annual precipitation of the region is 650 mm, and the average temperature is 17.0 °C in July and −6.7 °C in January. The duration of the growing season is typically 175–180 days from mid-April to October^53^.

Nine representative mature grey alder trees were selected for stem flux measurements at the FP, four at the CP (Supplementary Fig. S4). Soil fluxes were investigated close to each selected tree. The dominating ground vegetation at both plots was *Alnus incana* ((L.) Moench), *Filipendula ulmaria* ((L.) Maxim.), *Prunus padus* (L.), and *Rubus idaeus* (L.).

### Stem flux measurements

The tree fluxes were measured manually: five measurement sets in the pre-, six in the experimental and four in the post-experimental period. The representative trees were equipped with static closed tree stem chamber systems for stem flux measurements^28^. The chambers were installed at the bottom part of the tree (approximately 10 cm above the soil). In addition, a vertical profile of the stem fluxes (measurements at three stem heights of approximately 10, 80 and 170 cm above the ground) was studied in 6 and 2 trees at the FP and CP, respectively. The chambers were installed in June 2017 one month prior to the campaign. The rectangular shape stem chambers were made of transparent plastic containers, including removable airtight lids (Lock & Lock, South Korea). The bottom was cut and hot-glued with a neoprene band. The chambers were sealed with non-acid silicone to the smoothed stem surface and tested for airtightness. Two chambers per profile were set randomly across 180° and interconnected with tubes into one system (total volume of 0.00119 m³) covering 0.0108 m² of stem surface. A pump (Thomas, Germany, model 1410VD, 12 V) was used to homogenize the gas concentration prior to sampling. Chamber systems remained open between each sampling campaign. During 15 measurement campaigns, four gas samples (25 ml) were collected from each chamber system via septum in a 60 min interval: 0/60/120/180 min sequence (sampling time between 12:00 and 16:00) and stored in pre-evacuated (0.3 bar) 12 ml coated gas-tight vials (LabCo International, United Kingdom). The gas samples were analysed in the laboratory at University of Tartu within 2 weeks using gas chromatography (GC-2014; Shimadzu, Japan) equipped with an electron capture detector for detection of N_2_O and a flame ionization detector for CH_4_. The gas samples were injected automatically using Loftfield autosampler (Loftfield / Germany). For gas-chromatographical settings see Soosaar *et al*.^54^.

Fluxes were quantified on a linear approach according to change of CH_4_ and N_2_O concentrations in the chamber headspace over time, using the equation according to Livingston and Hutchison^55^.

### Soil flux measurements

Soil fluxes were measured using automatic dynamic chambers located close to each measurement tree and installed in June 2017. Nine chambers were situated at the FP, four at the CP. Every PVC made soil chamber covered a 0.16 m² soil surface, containing a volume of 0.032 m³. To avoid stratification of gas inside of the chamber, air with a constant flow rate 1.8 L/min was circulated within a closed loop between the chamber and gas analyzer unit during the measurements by a diaphragm pump. The air sample was taken from the top of the chamber headspace and pumped back by distributing it to each side of the chamber. For the measurements, the soil chambers were closed automatically for a duration of 9 minutes each. Flushing time of the whole system with ambient air between measurement periods was 1 minute. Thus, there were approximately 12 measurements per chamber per day. A Picarro G2508 (Picarro Inc., United States) gas analyzer using cavity ring-down spectroscopy (CRDS) technology was used to monitor CO_2_, CH_4_ and N_2_O gas concentrations in the frequency of approximately 1.17 measurements per second. The chambers were connected to the gas analyzer using a multiplexer.

### Flux calculations

The air temperature in the chamber was measured and used to convert the concentrations from ppm[v] to mg m^−3^ according to the ideal gas law before flux calculation^56^. Soil fluxes were calculated for a selected time window (150 seconds), after discarding initial 90 seconds to exclude the initial stabilisation period, using the linear model. The linear models is based on the assumption of a linear relationship between concentrations inside the chamber headspace and time. Fluxes were calculated using the equation, according to Livingston and Hutchison^55^.

To compare the contribution of soil and stems, the stem fluxes were up-scaled to hectare of ground area based on average tree diameter, stem surface area, tree density, and stand basal area estimated for each period and plot. A cylindric shape of tree stem was assumed. To estimate average stem emissions, fitted regression curves for different periods were made between the stem emissions and height of the measurements as previously done by Sjögersten *et al*.^57^. The regression model parameters are reported in Supplementary Table S2.

### Data quality check

Fluxes were quantified on a linear approach according to change of CO_2_, CH_4_ and N_2_O concentrations in the chamber headspace over time. A data quality control was applied based on R^2^ values of linear fit for CO_2_ measurements. When the R^2^ value for CO_2_ efflux was above 0.9, the conditions inside the chamber were applicable, and the calculations for both CH_4_ and N_2_O gases were also accepted in spite of their R^2^ values. Calculations with lower R^2^ value (R^2^ < 0.9) were removed from the database.

### Ancillary measurements

Automatic groundwater level data loggers (Hobo U20L-04, Onset Computer Corporation, USA) were installed in groundwater wells. Soil temperature (107, CAMPBELL SCIENTIFIC. INC, USA) and soil moisture sensors (ML3 ThetaProbe, Delta-T Devices, United Kingdom) were installed at 0–10 cm soil depth close to adjacent tree spots. During five campaigns (two in the pre-experimental period, two in experimental period, and one in post-experimental period) composite topsoil samples with soil corer at depth 0–10 cm were taken for physical and chemical analysis using standard methods^58^.

### Statistical analysis

Principal Component Analysis (PCA) was performed on real and imputed data sets of soil physicochemical and gas flux measurements using the R package ade4 v. 1.7–13^59^. For PCA, missing data patterns were identified and explored using the packages “naniar” v. 0.4.2^60^ and “VIM” v. 4.8.0^61^ (Supplementary Table S3). In addition, R package “missMDA” v. 1.14^62^ was applied to impute missing data values in case of PCA. Differences in PCA between the plots and periods were evaluated using PERMANOVA with 9999 permutations and pairwise comparisons were corrected with Bonferroni method, using the R package vegan v. 2.5-6^63^. To evaluate the significance of the differences between the plots and their periods with respect to physicochemical variables values, multivariate linear models were constructed, and models were tested after 9999 permutations using the anova function in R package mvabund v. 4.0.1^64^. Linear mixed-effects models (LMM) were applied to investigate differences in soil moisture and gas flux measurements between the control and flooded plots using the R method “lmer” incorporating both temporal (sampling days) and spatial (different trees) effects as random effects (v. 1.1–21)^65^, while p-values were calculated in order to confirm the significance of the relationships using the R package lmerTest v. 3.0-1^66^. The same mixed effects method was used to test statistical significance between different periodic groups of soil and stem fluxes. LMM was also used to detect differences between stem fluxes of different stem heights in different periods. For exploring LMMs fit, inspection of residuals patterns for the model was used as diagnostic tool^65,67^. To meet the analysis assumptions, rank transformation of the data was performed and LMM on the rank-transformed response variables are analysed and reported^68^. The regression models for stem heights and stem fluxes of different periods were estimated by nonlinear least squares, using the R function “nls.” R version 3.6.1 (R Development Core Team, 2019) was used to conduct all the statistical analyses. The significance level (alpha) considered for all the tests was 0.05.

## Supplementary information

Supplementary information.

## References

[1] Intergovernmental Panel on Climate Change. *Climate Change 2014: Mitigation of Climate Change: Working Group III Contribution to the IPCC Fifth Assessment Report*. *Working Group III Contribution to the Fifth Assessment Report of the Intergovernmental Panel on Climate Change*. 10.1017/CBO9781107415416 (2014).

[2] Saikawa E (2014). Global and regional emissions estimates for N2O. Atmos. Chem. Phys..

[3] Tian H (2016). The terrestrial biosphere as a net source of greenhouse gases to the atmosphere. Nature.

[4] Dalal RC, Allen DE (2008). Greenhouse gas fluxes from natural ecosystems. Aust. J. Bot..

[5] Covey Kristofer R., Megonigal J. Patrick (2019). Methane production and emissions in trees and forests. New Phytologist.

[6] Barba Josep, Bradford Mark A., Brewer Paul E., Bruhn Dan, Covey Kristofer, Haren Joost, Megonigal J. Patrick, Mikkelsen Teis Nørgaard, Pangala Sunitha R., Pihlatie Mari, Poulter Ben, Rivas‐Ubach Albert, Schadt Christopher W., Terazawa Kazuhiko, Warner Daniel L., Zhang Zhen, Vargas Rodrigo (2018). Methane emissions from tree stems: a new frontier in the global carbon cycle. New Phytologist.

[7] Megonigal JP, Guenther AB (2008). Methane emissions from upland forest soils and vegetation. Tree Physiol..

[8] Gauci V, Gowing DJG, Hornibrook ERC, Davis JM, Dise NB (2010). Woody stem methane emission in mature wetland alder trees. Atmos. Environ..

[9] Pangala SR, Moore S, Hornibrook ERC, Gauci V (2013). Trees are major conduits for methane egress from tropical forested wetlands. New Phytol..

[10] Smith Ka (2003). Exchange of greenhousegases between soil and atmosphere: interactions of soil physical factors and biological processes. Eur. J. Soil Sci..

[11] Rusch H, Rennenberg H (1998). Black alder (Alnus glutinosa (L.) Gaertn.) trees mediate methane and nitrous oxide emission from the soil to the atmosphere. Plant Soil.

[12] Machacova, K., Papen, H., Kreuzwieser, J. & Rennenberg, H. *Inundation strongly stimulates nitrous oxide emissions from stems of the upland tree Fagus sylvatica and the riparian tree Alnus glutinosa*. *Plant and Soil* vol. 364 (2013).

[13] Pangala SR, Hornibrook ERC, Gowing DJ, Gauci V (2015). The contribution of trees to ecosystem methane emissions in a temperate forested wetland. Glob. Chang. Biol..

[14] Terazawa K, Ishizuka S, Sakata T, Yamada K, Takahashi M (2007). Methane emissions from stems of Fraxinus mandshurica var. japonica trees in a floodplain forest. Soil Biol. Biochem..

[15] Aosaar J, Varik M, Uri V (2012). Biomass production potential of grey alder (Alnus incana (L.) Moench.) in Scandinavia and Eastern Europe: A review. Biomass and Bioenergy.

[16] Uri V, Lõhmus K, Kiviste A, Aosaar J (2009). The dynamics of biomass production in relation to foliar and root traits in a grey alder (Alnus incana (L.) Moench) plantation on abandoned agricultural land. Forestry.

[17] Uri, V., Tullus, H. & Lo, K. Uri (2001) Biomass production and nutrien accumulation in short-rotation grey alder.pdf. **161**, 169–179 (2002).

[18] Rytter L, Rytter RM (2016). Growth and carbon capture of grey alder (Alnus incana (L.) Moench.) under north European conditions - Estimates based on reported research. For. Ecol. Manage..

[19] Evans J (2006). Silviculture of Broadleaved Woodland. J. Appl. Ecol..

[20] Vogel CS, Curtis PS, Thomas RB (1997). Growth and nitrogen accretion of dinitrogen-fixing Alnus glutinosa (L.) Gaertn. under elevated carbon dioxide. Plant Ecol..

[21] Krzaklewski W, Pietrzykowski M, WoŚ B (2012). Survival and growth of alders (Alnus glutinosa (L.) Gaertn. and Alnus incana (L.) Moench) on fly ash technosols at different substrate improvement. Ecol. Eng..

[22] Rosenvald K (2011). Rhizosphere effect and fine-root morphological adaptations in a chronosequence of silver birch stands on reclaimed oil shale post-mining areas. Ecol. Eng..

[23] Šourková M, Frouz J, Šantrůčková H (2005). Accumulation of carbon, nitrogen and phosphorus during soil formation on alder spoil heaps after brown-coal mining, near Sokolov (Czech Republic). Geoderma.

[24] Roy S, Khasa DP, Greer CW (2007). Combining alders, frankiae, and mycorrhizae for the revegetation and remediation of contaminated ecosystems. Can. J. Bot..

[25] Huth, V. *et al*. The climate warming effect of a fen peat meadow with fluctuating water table is reduced by young alder trees. **21**, 1–18 (2018).

[26] *Biology, Controls and Models of Tree Volatile Organic Compound Emissions*. vol. 5 (Springer Netherlands, (2013).

[27] Maier M, Machacova K, Lang F, Svobodova K, Urban O (2018). Combining soil and tree-stem flux measurements and soil gas profiles to understand CH4 pathways in Fagus sylvatica forests. J. Plant Nutr. Soil Sci..

[28] Machacova K, Maier M, Svobodova K, Lang F, Urban O (2017). Cryptogamic stem covers may contribute to nitrous oxide consumption by mature beech trees. Sci. Rep..

[29] Niinemets Ü (2017). Environmental feedbacks in temperate aquatic ecosystems under global change: why do we need to consider chemical stressors?. Reg. Environ. Chang..

[30] Unger IM, Kennedy AC, Muzika R-M (2009). Flooding effects on soil microbial communities. Appl. Soil Ecol..

[31] Unger IM, Motavalli PP, Muzika R-M (2009). Changes in soil chemical properties with flooding: A field laboratory approach. Agric. Ecosyst. Environ..

[32] Lohila A (2010). Responses of N2O fluxes to temperature, water table and N deposition in a northern boreal fen. Eur. J. Soil Sci..

[33] Maljanen M (2017). The emissions of nitrous oxide and methane from natural soil temperature gradients in a volcanic area in southwest Iceland. Soil Biol. Biochem..

[34] Mer JL, Roger P, Provence D, Luminy D (2001). of methane by soils: A review. Archaea.

[35] Butterbach-Bahl, K., Baggs, E. M., Dannenmann, M., Kiese, R. & Zechmeister-Boltenstern, S. Nitrous oxide emissions from soils: How well do we understand the processes and their controls? *Philos. Trans. R. Soc. B Biol. Sci*. **368** (2013). 10.1098/rstb.2013.0122 PMC3682742 23713120

[36] Pärn J (2018). Nitrogen-rich organic soils under warm well-drained conditions are global nitrous oxide emission hotspots. Nat. Commun..

[37] Klemedtsson L, Svensson BH, Rosswall T (1988). Relationships between soil moisture content and nitrous oxide production during nitrification and denitrification. Biol. Fertil. Soils.

[38] Bateman EJ, Baggs EM (2005). Contributions of nitrification and denitrification to N2O emissions from soils at different water-filled pore space. Biol. Fertil. Soils.

[39] Klemedtsson L, Svensson BH, Rosswall T (1988). A method of selective inhibition to distinguish between nitrification and denitrification as sources of nitrous oxide in soil. Biol. Fertil. Soils.

[40] Davidson EA, Swank WT (1986). Environmental parameters regulating gaseous nitrogen losses from two forested ecosystems via nitrification and denitrification. Appl. Environ. Microbiol..

[41] Keppler F, Hamilton JTG, Braß M, Röckmann T (2006). Methane emissions from terrestrial plants under aerobic conditions. Nature.

[42] Terazawa K, Yamada K, Ohno Y, Sakata T, Ishizuka S (2015). Spatial and temporal variability in methane emissions from tree stems of Fraxinus mandshurica in a cool-temperate floodplain forest. Biogeochemistry.

[43] Mander Ü (2015). The impact of a pulsing groundwater table on greenhouse gas emissions in riparian grey alder stands. Environ. Sci. Pollut. Res..

[44] Rice AL (2010). Emissions of anaerobically produced methane by trees. Geophys. Res. Lett..

[45] Pitz SL, Megonigal JP, Chang CH, Szlavecz K (2018). Methane fluxes from tree stems and soils along a habitat gradient. Biogeochemistry.

[46] Machacova K (2016). Pinus sylvestris as a missing source of nitrous oxide and methane in boreal forest. Sci. Rep..

[47] Machacova K, Vainio E, Urban O, Pihlatie M (2019). Seasonal dynamics of stem N2O exchange follow the physiological activity of boreal trees. Nat. Commun..

[48] Trenberth KE (2011). Changes in precipitation with climate change. Clim. Res..

[49] Nicholls, R. J., Hoozemans, F. M. J. & Marchand, M. Increasing flood risk and wetland losses due to global sea-level rise: Regional and global analyses. *Glob. Environ. Chang*. **9** (1999).

[50] Semmler T, Jacob D (2004). Modeling extreme precipitation events - A climate change simulation for. Europe. Glob. Planet. Change.

[51] Blöschl G (2019). Changing climate both increases and decreases European river floods. Nature.

[52] Vargas R (2019). & Barba. J. Greenhouse Gas Fluxes From Tree Stems. Trends Plant Sci..

[53] Kupper P (2011). An experimental facility for free air humidity manipulation (FAHM) can alter water flux through deciduous tree canopy. Environ. Exp. Bot..

[54] Soosaar K (2011). Dynamics of gaseous nitrogen and carbon fluxes in riparian alder forests. Ecol. Eng..

[55] Livingston, G. P. & Hutchinson, G. L. Enclosure-based measurement of trace gas exchange: Applications and sources of error. in *Biogenic Trace Gases: Measuring Emissions from Soil and Water* (eds. Matson, P. A. & R.C., H.) 14–51 (Ed. Blackwell Publishing: Oxford, Unitel Kingdom (1995).

[56] Collier, S. M., Ruark, M. D., Oates, L. G., Jokela, W. E. & Dell, C. J. Measurement of greenhouse gas flux from agricultural soils using static chambers. *J. Vis. Exp*. 10.3791/52110 (2014). 10.3791/52110 PMC4827936 25146426

[57] Sjögersten Sofie, Siegenthaler Andy, Lopez Omar R., Aplin Paul, Turner Benjamin, Gauci Vincent (2019). Methane emissions from tree stems in neotropical peatlands. New Phytologist.

[58] Apha, Water Environment Federation & American Water Works Association. Standard Methods for the Examination of Water and Wastewater (Part 1000–3000). *Stand. Methods Exam. Water Wastewater* 733, doi: ISBN 9780875532356 (1999).

[59] Dray, S., Dufour, A. Ade4: Analysis of Ecological Data. *Explor. Euclidean Methods Environ. Sci*. **22** (2007).

[60] Tierney, N. *et al*. Package ‘ naniar’ R topics documented: (2019).

[61] Kowarik, A. & Templ, M. Imputation with the R package VIM. *J. Stat. Softw*. **74** (2016).

[62] Josse, J. & Husson, F. missMDA: A Package for Handling Missing Values in Multivariate Data Analysis. *J. Stat. Softw*. **70** (2016).

[63] Oksanen, J. *et al*. Package ‘ vegan’. 0–291 (2019).

[64] Wang Y, Naumann U, Wright ST, Warton DI (2012). Mvabund- an R package for model-based analysis of multivariate abundance data. Methods Ecol. Evol..

[65] Bates, D., Mächler, M., Bolker, B. & Walker, S. Fitting Linear Mixed-Effects Models Using lme4. *J. Stat. Softw*. **67** (2015).

[66] Kuznetsova, A., Brockhoff, P. B. & Christensen, R. H. B. lmerTest Package: Tests in Linear Mixed Effects Models. *J. Stat. Softw*. **82** (2017).

[67] Harrison, X. A. *et al*. A brief introduction to mixed effects modelling and multi-model inference in ecology. *PeerJ* **2018**, 1–32 (2018). 10.7717/peerj.4794 PMC5970551 29844961

[68] Quian, S. S. *Environmental and Ecological Statistics with R*. (ISBN9781315370262). 10.1201/9781315370262 2016.

