# Supplementary material for: Short-term flooding increases CH4 and N2O emissions from trees in a riparian forest soil-stem continuum
Source: Sci Rep. 2020 Feb 21;10:3204. doi: 10.1038/s41598-020-60058-7 (PMC7035275; doi:10.1038/s41598-020-60058-7)
Supplement: Supplementary file 1 — Supplementary information. [file 41598_2020_60058_MOESM1_ESM.docx]

**Short-term flooding increases CH_4_ and N_2_O emissions from trees in a riparian forest soil-stem continuum**

AUTHORS

Thomas Schindler^1, 2*^, Ülo Mander^1^, Katerina Machacova^2^, Mikk Espenberg^1^, Dmitrii Krasnov^3^, Jordi Escuer-Gatius^4^, Gert Veber^1^, Jaan Pärn^1^, Kaido Soosaar^1^

^1^ Department of Geography, Institute of Ecology & Earth Sciences, University of Tartu, Tartu, Estonia ^2^ Department of Ecosystem Trace Gas Exchange, Global Change Research Institute CAS, Brno, Czech Republic ^3^ Department of Plant Physiology, Estonian University of Life Sciences, Tartu, Estonia ^4^ Chair of Soil Science, Estonian University of Life Sciences, Tartu, Estonia

*Correspondence: [*thomas.schindler@ut.ee*](mailto:thomas.schindler@ut.ee) *(T. Schindler)*

# Supplementary Information

Supplementary Table S1. Linear mixed-effects models were applied to investigate differences in soil moisture and gas flux measurements between the control (CP) and flooding plots (FP) in different periods. Abbreviations: not significant (ns), CH_4_/N_2_O_Stem0/80/170(Soil denote measurements at three stem heights of 0.1, 80 and 170 cm above ground respectively from soil

| Parameter | Abbreviations | Pre-experiment (CP vs FP) | | | Experiment (CP vs FP) | | | Post-experiment (CP vs FP) | | |
| --- | --- | --- | --- | --- | --- | --- | --- | --- | --- | --- |
|  |  | n | t value | p value | n | t value | p value | n | t value | p value |
| Soil water content | SWC | 60 |  | ns | 84 | 12.97 | p<0.001 | 48 | 2.69 | p<0.05 |
| CH_4_ | CH_4__Stem170 | 48 |  | ns | 53 | 7.28 | p<0.001 | 32 | 7.10 | p<0.001 |
|  | CH_4__Stem80 | 48 | 2.21 | p<0.05 | 53 | 7.06 | p<0.001 | 32 | 17.49 | p<0.001 |
|  | CH_4__Stem0 | 72 | 3.47 | p<0.001 | 79 | 9.98 | p<0.001 | 48 | 7.28 | p<0.001 |
|  | CH_4__Soil | 55 |  | ns | 77 | 2.60 | p<0.05 | 41 | 4.46 | p<0.001 |
| N_2_O | N_2_O_Stem170 | 48 |  | ns | 53 |  | ns | 32 | -4.59 | p<0.001 |
|  | N_2_O_Stem80 | 48 |  | ns | 53 |  | ns | 32 | -5.53 | p<0.001 |
|  | N_2_O_Stem0 | 72 | -6.68 | p<0.001 | 79 | -8.88 | p<0.001 | 48 | -10.24 | p<0.001 |
|  | N_2_O_Soil | 55 |  | ns | 78 |  | ns | 42 |  | ns |

Supplementary Table S2. The regression models for stem heights and stem fluxes of different plots and periods. The best fit model was an exponential model f = a*exp(-b*x). Abbreviations: control plot (CP), flooding plot (FP)

| Gas | Site | Period | a | b |
| --- | --- | --- | --- | --- |
| CH_4_ | CP | Pre-Experiment | 0.72 (p=0.2) | 0.0014 (p=0.8) |
|  |  | Experiment | 0.84 (p<0.05) | 0.0171 (p=0.5) |
|  |  | Post-Experiment | 9.07 (p=0.9) | 0.1607 (p=1.0) |
|  | FP | Pre-Experiment | 4.82 (p<0.001) | 0.0136 (p=0.08) |
|  |  | Experiment | 84.87 (p<0.001) | 0.0148 (p=0.07) |
|  |  | Post-Experiment | 407.87 (p<0.001) | 0.0091 (p<0.05) |
| N_2_O | CP | Pre-Experiment | 10.34 (p=0.9) | 0.1295 (p=0.9) |
|  |  | Experiment | 13.25 (p<0.001) | 0.0478 (p=0.7) |
|  |  | Post-Experiment | 78.68 (p<0.001) | 0.0318 (p=0.3) |
|  | FP | Pre-Experiment | 6.20 (p<0.001) | 0.0123 (p<0.05) |
|  |  | Experiment | 15.16 (p<0.001) | 0.0113 (p<0.05) |
|  |  | Post-Experiment | 2.19 (p<0.001) | 0.0227 (p=0.3) |

*Supplementary Table S3. Percent of missing data points, which were imputed for the principal components analysis (PCA). Percentage of missing value in the whole data was 9.77%.* *Abbreviations: CH4/N2O_Stem0/80/170(Soil denote measurements at three stem heights of 0.1, 80 and 170 cm above ground respectively from soil*

| Parameter | Total data points | Observed data points | Proportion missing (%) |
| --- | --- | --- | --- |
| CH_4__Stem170 | 55 | 35 | 0.36 |
| N_2_O_Stem170 | 55 | 35 | 0.36 |
| CH_4__Stem80 | 55 | 35 | 0.36 |
| N_2_O_Stem80 | 55 | 35 | 0.36 |
| N_2_O_Soil | 55 | 50 | 0.09 |
| CH_4__Soil | 55 | 54 | 0.02 |
| N | 55 | 55 | 0 |
| NH_4_ | 55 | 55 | 0 |
| P | 55 | 55 | 0 |
| Organic matter | 55 | 55 | 0 |
| NO_3_ | 55 | 55 | 0 |
| pH_KCl_ | 55 | 55 | 0 |
| Soil moisture | 55 | 55 | 0 |
| Soil temperature | 55 | 55 | 0 |
| CH_4__Stem0 | 55 | 55 | 0 |
| N_2_O_Stem0 | 55 | 55 | 0 |

**
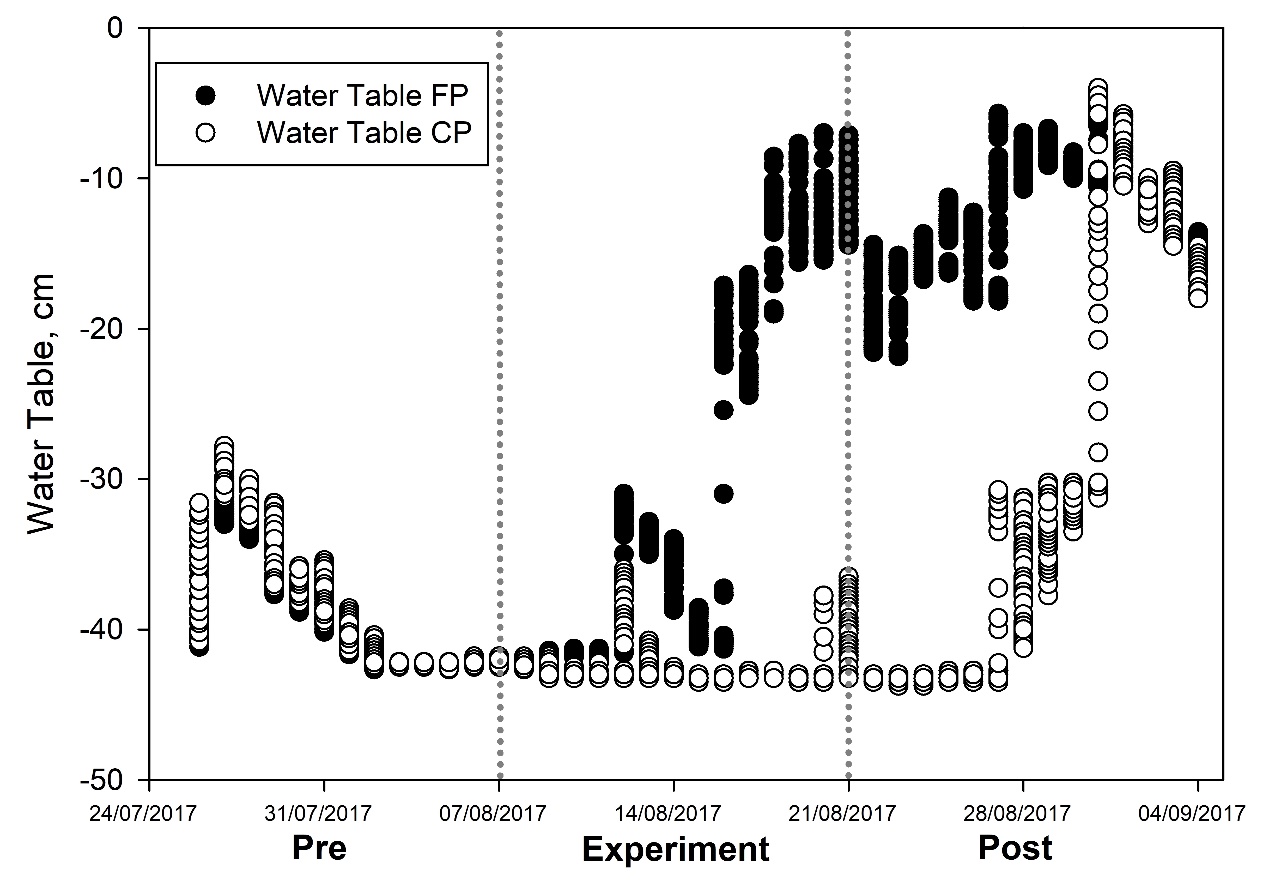
**

Supplementary Figure S1: Water Table mean values, Flooded and Control Plot (n=48 per day)


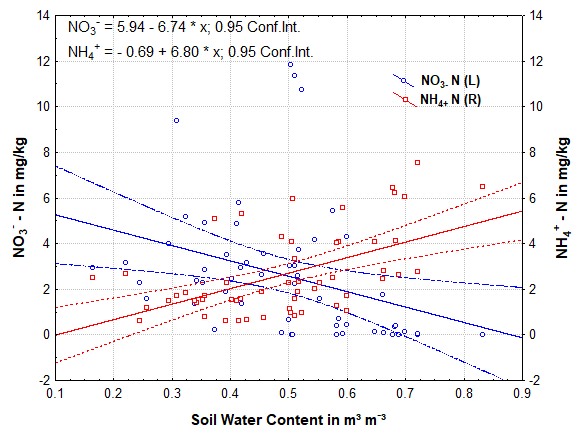


Supplementary Figure S2. Changes in nitrate (NO_3_^-^) and ammonia (NH_4_^+^) concentration (mg/kg) in the soil versus dynamics of soil water content at flooded plot.


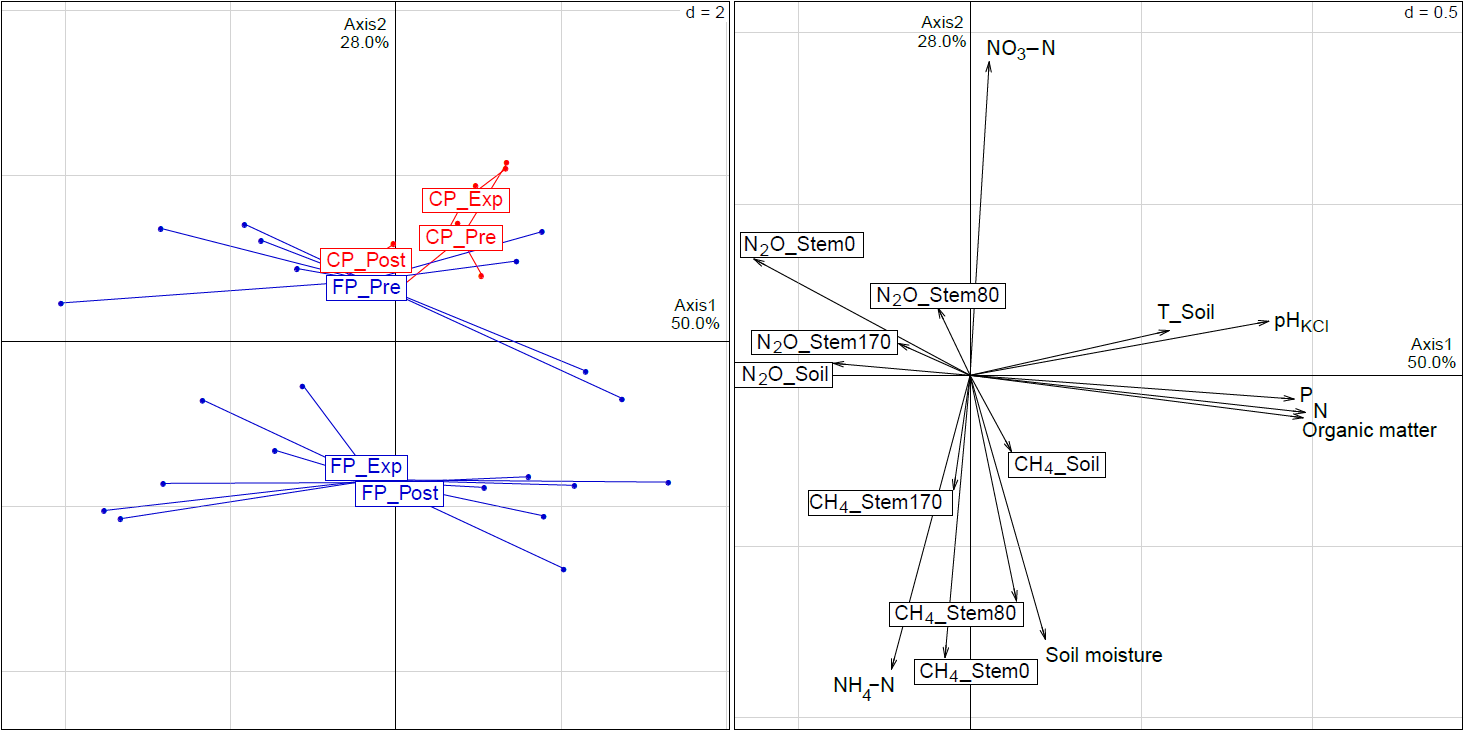


Supplementary Figure S3. Characteristics of physico-chemical and gas flux parameters in the flooded (FP) and control plots (CP) of pre-experimental (Pre), experimental (Exp), and post-experimental (Post) periods. The principal components analysis (PCA) is based on real data set (n = 31). Abbreviations: T_Soil – soil temperature; Stem0, Stem80, and Stem170 denote measurements at three stem heights of 10, 80 and 170 cm above the ground.


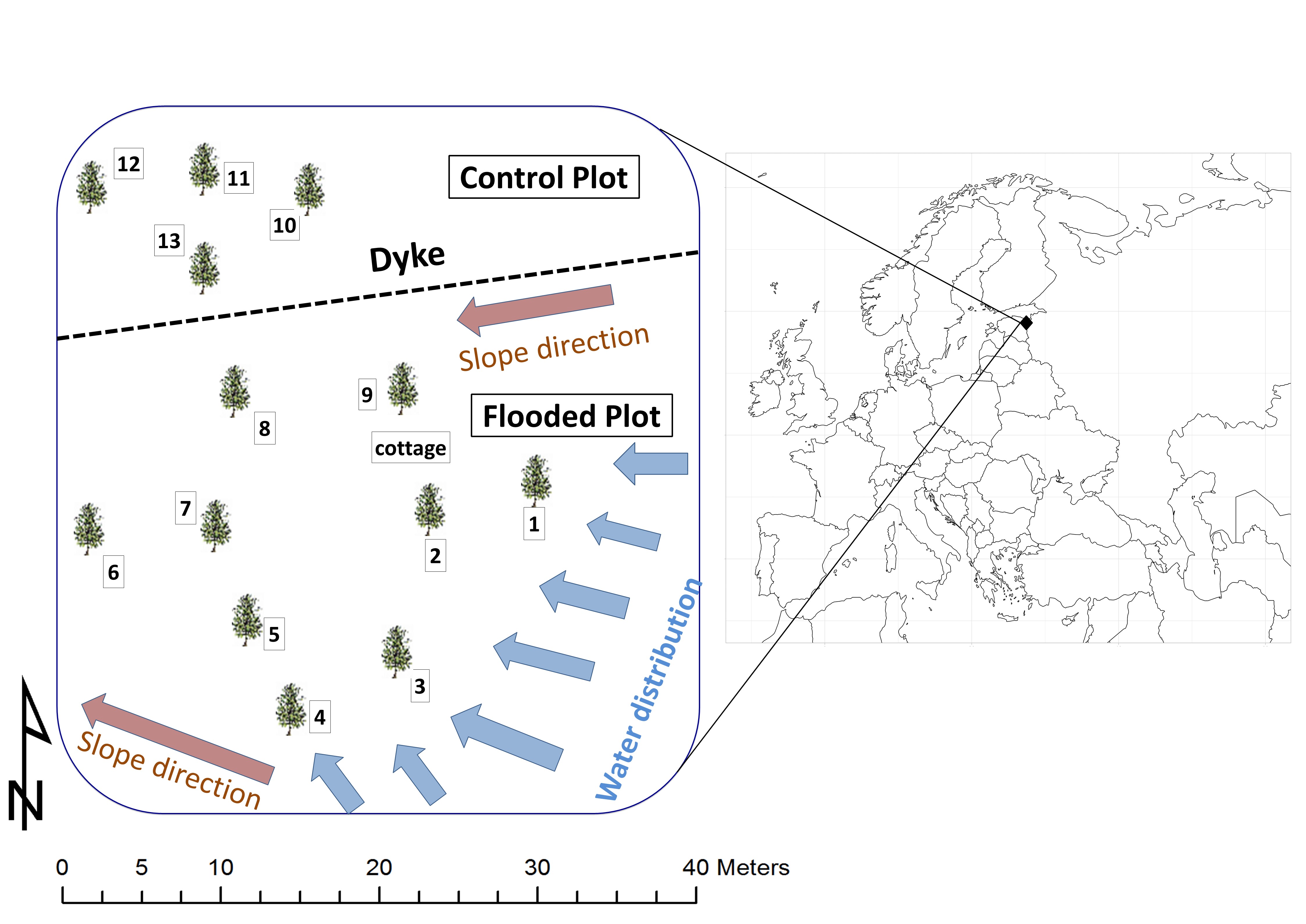


Supplementary Figure S4. Location and schematic view of the study site. The flooded and control plots are separated by a 1 m high dyke and isolated regarding the groundwater flow. Numbers 1–13 indicate paired soil and stem chambers. Map was created with ggplot2 version 3.2.1 package (https://ggplot2.tidyverse.org) for R 3.6.1 (R Development Core Team 2019)
